# The role of hormonal and nutritional biomarkers in predicting immune checkpoint inhibitor efficacy: current limitations and future prospects

**DOI:** 10.3389/fimmu.2026.1857147

**Published:** 2026-08-13

**Authors:** Ruichen Yuan, Yuejiao Lan, Mingda Wu, Xiaodan Lu

**Affiliations:** 1 College of Integrative Chinese and Western Medicine, Changchun University of Chinese Medicine, Changchun, Jilin, China; 2 Precision Molecular Medicine Center, Jilin Province People’s Hospital, Changchun, Jilin, China

**Keywords:** endocrine biomarkers, host physiology, immune checkpoint inhibitors, nutritional biomarkers, precision immunotherapy

## Abstract

Immune checkpoint inhibitors (ICIs) have transformed cancer treatment, yet durable benefit remains limited to selected patients. Established biomarkers, including PD-L1 expression, tumor mutational burden, and microsatellite instability, remain predominantly tumor-centered and incompletely capture inter-individual variability. In this Perspective, we highlight systemic host physiology as an underappreciated determinant of the tumor microenvironment, ICI efficacy, and immune-related toxicity. We discuss how the hypothalamic–pituitary–adrenal axis, thyroid function, insulin signaling, nutritional status, micronutrients such as vitamin D and iron, emerging metabolic modulators, body composition, and the gut microbiome may influence T-cell activation, exhaustion, and myeloid-cell function. We also examine unresolved controversies, including the obesity paradox, the potential risk of GLP-1 receptor agonist-associated sarcopenia, and the lack of standardized biomarker thresholds and sampling protocols. To facilitate clinical translation, we outline a conceptual, hypothesis-generating spatiotemporal management framework integrating pre-treatment host profiling, peri-treatment metabolic and nutritional modulation, and post-treatment dynamic monitoring. Composite indices such as the Prognostic Immune and Nutritional Index, together with precise body-composition assessment, may improve risk stratification. However, host-directed interventions should be interpreted cautiously because current evidence remains heterogeneous and largely observational or preclinical. Prospective, multidisciplinary studies are required to validate these concepts and determine whether host-directed strategies can improve ICI efficacy while limiting immune-related toxicity.

## 1 Introduction

According to the 2023 Global Burden of Disease study, cancer remains the second leading cause of mortality globally. In 2023, approximately 18.5 million individuals were diagnosed worldwide, with 10.4 million deaths; projections for 2050 anticipate an annual incidence approaching 30.5 million and 18.6 million deaths. Largely modifiable environmental, behavioral, and metabolic risk factors are implicated in nearly 41.7% of cancer-related deaths (1), underscoring the need for targeted and efficient therapeutic strategies.

Over the past decade, oncology has undergone a marked paradigm shift driven by immunotherapy, and immune checkpoint inhibitors (ICIs) have redefined treatment algorithms for advanced malignancies (2). By targeting inhibitory axes, specifically CTLA-4, PD-1, and its ligand PD-L1, these monoclonal antibodies relieve tumor-induced immune suppression, reactivating exhausted T cells and enabling durable responses (3). Improvements in long-term survival have since been documented across diverse solid tumors, including melanoma, non-small cell lung cancer (NSCLC), renal cell carcinoma (RCC), and gastric malignancies (4–7).

Despite these advances, the efficacy of immune checkpoint blockade remains heterogeneous. Durable benefit from ICI monotherapy is achieved by only a fraction of patients, typically between 20% and 40%, while the majority experience primary resistance or acquired resistance after an initial response (7, 8).

Toxicity presents an equally difficult challenge. ICIs frequently cause a distinct spectrum of autoimmune-like side effects, termed immune-related adverse events (irAEs), which can affect virtually any organ system and occasionally become life-threatening (9). This risk-benefit profile makes it a central task in immuno-oncology to predict toxicity while identifying the patients biologically predisposed to favorable responses.

To address these challenges, research has increasingly turned toward reliable predictive biomarkers. To date, only a few have secured U.S. Food and Drug Administration (FDA) approval: PD-L1 expression, mismatch repair deficiency (dMMR) or high microsatellite instability (MSI-H), and tumor mutational burden (TMB) (10).

In routine practice, however, these tumor-intrinsic metrics have substantial limitations. Standardization of PD-L1 assessment remains inadequate, and the molecule shows considerable spatiotemporal heterogeneity, so its reliability as a predictive tool varies with the malignancy and therapeutic protocol (7). Similar caveats apply to genomic markers: while TMB and MSI-H offer prognostic value in specific contexts, analyses of cohorts exceeding 100,000 patients across a hundred cancer histologies show that elevated TMB is present in only a small fraction of the pan-cancer population (11).

Determining these genomic profiles also frequently requires tissue-based next-generation sequencing (NGS), yet adequate biopsies are often invasive and difficult to obtain, and technical and financial barriers further hinder routine implementation. These limitations point to a deeper reality: therapeutic success is rarely governed solely by the tumor’s intrinsic properties, but is also shaped by the systemic physiological state of the host (12).

This forms the central thesis of the present review. Research perspectives must broaden beyond the immediate tumor microenvironment (TME) to encompass the host’s metabolic and endocrine landscape. Emerging data indicate that immune homeostasis is regulated by host-derived variables, particularly baseline nutritional status and circulating hormone levels, which govern immune-cell development, differentiation, and effector function and thereby help shape the efficacy of ICI regimens (13).

A recent prospective observational study (STRESS-LUNG-1) in patients with advanced NSCLC provided clinical evidence consistent with this association. In that study, higher baseline emotional distress, rather than a predefined cortisol threshold, was the primary exposure: patients with pretreatment distress had a shorter median progression-free survival (PFS) (7.9 versus 15.5 months; HR = 1.73) and a lower objective response rate (ORR). Exploratory biomarker analyses in the same cohort found that elevated blood cortisol accompanied emotional distress and correlated with these adverse outcomes, supporting a neuroendocrine link rather than establishing cortisol as an independent predictive marker (14). Similar systemic vulnerabilities were highlighted by an extensive multicenter analysis comprising 1,791 individuals. Within this cohort, abbreviated overall survival (OS) was independently associated with deterioration of the patient’s baseline nutritional status. This physiological decline was most notably characterized by an increased density of subcutaneous fat alongside pronounced skeletal muscle wasting (15).

Guided by these insights, this review evaluates key nutritional and endocrine biomarkers, focusing on iron parameters, vitamin D, thyroid hormones, and serum cortisol. We analyze the pathways through which these systemic variables modulate ICI efficacy, assess their prognostic utility and the barriers to clinical integration, and outline future directions, offering a conceptual framework to support individualized, precision-driven immunotherapy.

## 2 Synthesis of current evidence

Anti-tumor immunity is a complex cascade shaped by the interplay among malignant cells, immune effectors, and the host’s systemic milieu (16). Although the TME remains the primary site of immune engagement, the systemic endocrine and metabolic landscape sets the basal functional competence of the immune system. The following sections review the mechanisms through which key hormonal and nutritional factors modulate ICI efficacy.

### 2.1 Endocrine regulation of anti-tumor immunity

#### 2.1.1 Glucocorticoids and immunosuppression

Acting as the primary neuroendocrine interface, the hypothalamic-pituitary-adrenal (HPA) axis orchestrates the host’s fundamental response to physiological and psychological stress (17). Within this pathway, glucocorticoids (GCs) serve as the dominant effectors, with cortisol, in particular, operating as a potent endogenous immunosuppressant (18).

Chronic hyperactivation of the HPA axis is routinely documented among oncology populations, typically manifesting as persistently elevated serum cortisol. The etiology of this phenomenon is multifactorial. The sheer metabolic demand of the tumor burden imposes profound physiological stress, which is systematically compounded by the aggressive nature of standard clinical interventions, including surgery, radiation, and chemotherapy. Furthermore, the profound psychological trauma inextricably linked to cancer diagnosis and long-term management significantly amplifies this continuous stress cascade (19, 20).

Evidence from human lung carcinoma cell lines and tissue samples indicates that some tumors can synthesize immunoregulatory glucocorticoids (21). These findings support tumor-associated steroidogenesis as a plausible contributor to local immune regulation; however, they do not establish that tumors systematically control this pathway or that it represents a dominant mechanism of immune escape across cancer types.

Beyond *de novo* synthesis, preclinical studies in murine tumor models indicate that tumor-cell 11β-hydroxysteroid dehydrogenase type 1 (11β-HSD1) can regenerate active glucocorticoids from inactive metabolites, thereby enhancing local glucocorticoid signaling and Treg-mediated immunosuppression (22). Whether this pathway operates to a comparable extent in human tumors, materially increases intratumoral cortisol concentrations, or influences clinical responses to ICIs remains to be determined.

On the basis of these observations, we propose a putative “Dual Endocrine Suppression Hypothesis.” We hypothesize that systemic HPA-axis activation and tumor-associated glucocorticoid synthesis or recycling may converge to reinforce an immunosuppressive TME. In this conceptual model, systemic stress would provide a background immunosuppressive signal, whereas local glucocorticoid metabolism could further limit anti-tumor immune activity. This proposed interaction should be regarded as a hypothesis rather than an established mechanism, because direct evidence for coordinated systemic-local synergy in patients is currently lacking.

The molecular machinery underlying this suppression is intricate. GCs bind cytoplasmic glucocorticoid receptors (GRs), triggering nuclear translocation of the complex, which then disables essential transcription factors, most notably nuclear factor-kappa B (NF-κB) and activator protein-1 (AP-1) (23, 24). Because interleukin-2 (IL-2) production within the T-cell receptor (TCR) cascade depends on these factors, persistent GR activation impairs T-cell proliferation, activation, and effector function (25, 26).

Prolonged exposure to these GCs exerts detrimental effects on overall immune competence. Specifically, both interferon-γ (IFN-γ) secretion and inherent cytotoxic capabilities are severely compromised (27, 28). Furthermore, GCs actively induce thymic involution, which drastically curtails the peripheral generation and output of naïve T cells (29). In the context of immune checkpoint blockade, an additional critical resistance mechanism has emerged: GCs directly upregulate the inhibitory receptor LAG-3 on macrophages and CD4+ T cells, a molecular alteration that potently blunts ICI efficacy (30).

Beyond these direct effects, recent work has clarified how GCs remodel the innate immune landscape to foster an immunosuppressive metastatic niche. Chronic stress, via systemic GC release, alters neutrophil behavior and promotes neutrophil extracellular traps (NETs) (31). These DNA-histone complexes shield the TME, impairing T-cell-mediated immunosurveillance and accelerating metastasis, and by limiting CD8+ T-cell access to malignant cells they contribute to primary resistance to ICI therapy (31).

The maturation and function of antigen-presenting cells (APCs), particularly dendritic cells (DCs), are similarly impaired (32). GCs downregulate surface MHC and the costimulatory molecules CD80 and CD86, disrupting naïve T-cell priming (33), in part through upregulation of the Tsc22d3 and acyl-CoA-binding protein (ACBP/DBI) axis, which disrupts type I interferon signaling (34). The DC population is thereby driven toward a tolerogenic phenotype (35).

Elevated GC levels also expand and activate regulatory T cells (Tregs) (36). Intratumoral GR activation increases secretion of immunosuppressive cytokines (G-CSF, M-CSF, TGF-β2, CXCL2), driving MDSC infiltration and Treg expansion (37), while polarizing macrophages toward a pro-tumorigenic M2 phenotype and suppressing natural killer (NK) cells (38). These populations inhibit effector T-cell activity through several complementary mechanisms (39).

Observational clinical evidence indicates that elevated baseline cortisol levels prior to ICI therapy are associated with a poorer prognosis. This correlation has been observed across various solid tumors, including melanoma, NSCLC, and RCC, as well as hematological malignancies (13). In these cohorts, higher cortisol, and the related state of pretreatment emotional distress, has been associated with diminished ORR and shorter PFS and OS (13, 14). Within specific microenvironments, such as ovarian cancer ascites, chronic stress-induced cortisol levels correlate negatively with pro-inflammatory cytokines and drive T-cell exhaustion alongside rapid disease progression (40).

In summary, the proposed “dual endocrine suppression” network, if operative, could undermine the anti-tumor immune foundation through coordinated systemic and local signaling. By antagonizing the immunostimulatory effects of ICIs at multiple levels, such a network might contribute to reduced therapeutic efficacy. We emphasize, however, that this integrated model is a hypothesis and has not yet been directly validated in patients.

#### 2.1.2 Thyroid hormones and immune homeostasis

The basal metabolic rate is regulated by thyroid hormones, which also serve as important mediators of immune homeostasis (41). Recent clinical evaluations have described a complex relationship linking a patient’s baseline thyroid status to the clinical efficacy of ICI therapy (42). An important clinical distinction must be drawn here: inherent, pre-treatment thyroid dysfunction and ICI-induced destructive thyroiditis represent distinct pathophysiological phenomena that should not be conflated.

Hypothyroidism present before immunotherapy typically reflects a state of host metabolic depression, which is associated with inferior survival trajectories (13). Across multiple large cohort studies, diminished ORRs, together with shorter PFS and OS, correlate significantly with baseline thyroid hormone deficiency (13).

Mechanistically, thyroid hormones have been reported to augment antigen presentation by DCs and to promote T-cell differentiation into effector phenotypes. Conversely, baseline thyroid insufficiency may reduce the bioenergetic fitness of T cells and impair their anti-tumor function. These host-derived metabolic constraints could therefore limit ICI efficacy, although this rationale is derived largely from preclinical and mechanistic data rather than from prospective clinical trials.

In contrast, patients who develop thyroid-related irAEs, such as thyroiditis, during ICI therapy frequently exhibit superior clinical outcomes (43). These events have been interpreted as a marker of on-target systemic immune activation rather than as mere fluctuations in thyroid function (44).

A recent comprehensive meta-analysis has illuminated a profound clinical connection: patients with malignancies, including NSCLC, who experience ICI-induced thyroid dysfunction demonstrate significantly prolonged OS and PFS. This survival advantage becomes exceptionally pronounced during the overt thyrotoxicosis phase (45). This physiological phenomenon remains highly consistent across distinct global demographics, encompassing both Asian and non-Asian cohorts. For example, in Asian populations, the hazard ratio (HR) for OS decreased to 0.53. During the acute overt thyrotoxicosis phase, the reported mortality-risk reduction is more pronounced, reaching an HR of 0.28 (45). However, these survival associations should be interpreted with caution. Treatment-emergent thyroid dysfunction is particularly susceptible to immortal-time bias, because patients must remain on therapy long enough to develop and have the event recorded; the association is further confounded by longer treatment duration and by more intensive endocrine surveillance in patients who respond. Time-dependent and landmark analyses within prospective cohorts are therefore required before treatment-emergent thyroid dysfunction can be regarded as a genuinely predictive, rather than prognostic, biomarker.

The biological mechanisms underlying these parallel outcomes are closely linked. Fundamentally, these events reflect systemic immune activation. Driven by peripheral infiltration of memory CD8+ T cells and expansion of T-cell clones, this heightened immune response concurrently targets normal thyroid architecture and eliminates disseminated malignant cells.

This crossover between immune homeostasis and thyroid biology highlights two predictive molecular markers with clinical potential: thyroglobulin antibodies (TGAb) and thyroid peroxidase antibodies (TPOAb). The clinical utility of these baseline serological profiles has been established by prospective cohort investigations. Abnormal pre-treatment levels of TGAb and TPOAb not only independently predict future ICI-mediated thyroid injury but are also associated with favorable long-term therapeutic outcomes (46, 47).

To integrate these observations, we propose, as a conceptual and hypothesis-generating framework rather than a validated tool, a Spatiotemporal Heterogeneity Predictive Model for thyroid function that would combine baseline screening with on-treatment monitoring. In this model, pre-treatment high-sensitivity TGAb and TPOAb could help flag potential exceptional responders and provide early warning of endocrine toxicity, while the on-treatment emergence of overt thyrotoxicosis could serve as a dynamic marker to guide supportive care. The framework is intended to link efficacy prediction with toxicity management, but it remains speculative and requires prospective validation.

#### 2.1.3 Additional endocrine axes

Sex hormones (estrogens and androgens) and the insulin/insulin-like growth factor-1 (IGF-1) axes exert considerable influence over the immune system (48). Androgens generally act as systemic immunosuppressants, compromising anti-tumor defenses by dysregulating the maturation, function, and viability of immune effector cells (49). Malignant tissues also exploit the local generation and processing of steroid hormones, including androgens, so that these local hormonal gradients help maintain an immunosuppressive TME (50).

Estrogen, by contrast, acts through context-dependent pathways, driving either immunostimulatory or immunosuppressive outcomes depending on local concentration, target cell lineage, and immunological milieu (51, 52). Both estrogen and progesterone frequently support systemic immune tolerance through induction of regulatory T-cell (Treg) differentiation and upregulation of immunosuppressive cytokines (53).

This regulation also extends into glucose homeostasis. Chronic low-grade inflammation is closely linked with hyperinsulinemia and insulin resistance (54), which compromise T-cell plasticity and metabolic fitness and blunt ICI efficacy (13). Accordingly, patients with pre-treatment insulin resistance consistently show inferior outcomes—diminished ORR and shorter PFS and OS—driven by accelerated T-cell exhaustion, expansion of myeloid-derived suppressive lineages, and a hostile microenvironment (13).

Immunity is also governed by adipokines, notably leptin and adiponectin, which influence immune-cell proliferation, differentiation, and cytokine output (55, 56). Host body composition, from cachexia to obesity, is thus a key determinant of efficacy (57). Moderate obesity occasionally predicts superior benefit: higher body mass index (BMI) correlates with improved survival in melanoma and NSCLC treated with ICIs (58, 59), possibly reflecting adipose reserves, leptin-mediated modulation, and better tolerance. Yet this “obesity paradox” may not be true protection, appearing driven by collider stratification bias, reverse causation from weight loss, and residual confounding (60). BMI also cannot distinguish visceral adiposity from muscle mass, and sarcopenic obesity confers no benefit (15, 61). Relying on BMI alone therefore has clear limitations.

### 2.2 Impact of nutritional status on anti-tumor immunity

Nutritional homeostasis supplies the biochemical substrates required for immune surveillance and effector function. Oncology patients frequently present with malnutrition, a systemic deficit that undermines quality of life and anti-tumor immune competence.

#### 2.2.1 Protein-energy malnutrition and immune dysfunction

Protein-energy malnutrition (PEM) is common in advanced malignancies, with hypoalbuminemia as its core serological hallmark (62). Albumin sustains plasma oncotic pressure and transports ligands such as fatty acids, drugs, and hormones; its circulating levels also mirror both the host’s nutritional reserves and the burden of systemic inflammation (63, 64).

Low pre-treatment serum albumin is associated with shorter PFS and OS following ICI therapy (65, 66), and a recent cohort study linked hypoalbuminemia to diminished ORR and inferior survival in hematological malignancies (13).

Mechanistically, PEM deprives immune cells, particularly lymphocytes, of essential amino acids, impairing proliferation and differentiation and hindering synthesis of effector proteins such as granzymes and perforin, thereby blunting CD8+ T-cell cytotoxicity (67). These disruptions also compromise core metabolic hubs, principally the IFN-γ and mTOR pathways, promoting myeloid-driven suppression and accelerating T-cell exhaustion.

Severe nutritional deficits rarely occur in isolation; they are coupled with the chronic inflammation of cancer cachexia, hallmarked by persistent pro-inflammatory cytokines, chiefly tumor necrosis factor-α (TNF-α) and interleukin-6 (IL-6). This inflammatory milieu shifts host metabolism, facilitating MDSC expansion and exacerbating T-cell exhaustion, and together these processes destabilize the anti-tumor immune network (68).

The significance of these host-derived variables is particularly evident in severe irAEs, notably ICI-induced colitis. Recent analyses identify elevated serum IL-6, at baseline or symptom onset, as an independent predictor of steroid-refractory disease (69), indicating that baseline inflammation and nutritional deterioration jointly antagonize therapeutic intervention.

Recognizing the limitations of isolated biomarkers, clinical strategies have increasingly evolved toward the implementation of integrated prognostic frameworks. Scoring systems such as the Prognostic Nutritional Index (PNI) and the Glasgow Prognostic Score (GPS) were specifically developed for this purpose. By synthesizing systemic inflammatory markers with serum albumin metrics, these composite tools achieve prognostic accuracy and clinical utility that exceeds single-parameter assessments (13, 70). Specifically, the PINI discussed herein refers to the Prognostic Immune and Nutritional Index, which is calculated as [Albumin (g/dL) × 0.9] − [Absolute Monocyte Count (/μL) × 0.0007] (71). This index must be distinguished from the traditional Prognostic Inflammatory and Nutritional Index, as its immune component, derived from the monocyte count, provides unique pathophysiological insights into the precursor status of MDSCs. A strong consensus in current research advocates for the routine integration of these composite scoring panels to accurately quantify overarching host health. This holistic physiological assessment is indispensable for optimizing individualized immunotherapeutic regimens, particularly when navigating the clinical complexities of refractory or relapsed malignancies (13).

#### 2.2.2 Micronutrients and immunomodulation

Immune regulation depends on basal biochemical homeostasis, within which host micronutrients serve as the primary source of essential signaling molecules and enzymatic cofactors.

Vitamin D: Its immunomodulatory role was historically characterized by broad anti-inflammatory effects, such as suppression of pro-inflammatory cytokines and promotion of protective mediators like IL-10 (72–74). Within the TME, however, vitamin D exerts context-dependent, pleiotropic effects that shape ICI efficacy (75). Its receptor (VDR) is expressed across immune lineages, including DCs, macrophages, and B and T lymphocytes (76), and immunomodulation is achieved predominantly when vitamin D engages these receptors.

Within the TME, vitamin D acts across stages of T-cell development. During priming, naive T cells show very low phospholipase C-gamma1 (PLC-γ1) expression and blunted T-cell antigen receptor (TCR) responsiveness (77); initial TCR triggering via the alternative p38 MAPK pathway then induces VDR expression (77). Engagement of this receptor drives an approximately 75-fold upregulation of PLC-γ1, a step required for classical TCR signaling, calcium mobilization, and activation of naive T cells (77).

Beyond activation, the active metabolite 1α,25-dihydroxyvitamin D3 (1α,25(OH)2D3) reverses effector T-cell exhaustion within the immunosuppressive TME (78). Under TCR stimulation, it drives epigenetic reprogramming in CD8+ and Vγ9Vδ2+ T cells (78): promoting DNA methylation of the Pdcd1 promoter to downregulate PD-1, TIM-3, and TIGIT, while enhancing H3K27 acetylation at the Cd28 promoter to upregulate CD28 (78). This facilitates Ca2+ influx and restores Th1 cytokines (IFN-γ and TNF-α) within exhausted T cells, supporting anti-tumor cytotoxicity (78).

Paradoxically, vitamin D can also be immunosuppressive under different conditions (75). In obesity-associated insulin resistance, 1α,25(OH)2D3-VDR signaling promotes fructose-1,6-bisphosphatase 1 (FBP1) expression, suppressing glycolysis in γδ T cells (75) and inhibiting Akt/p38 MAPK phosphorylation, which reduces IFN-γ and TNF-α upon PMA and ionomycin stimulation (75). Vitamin D thus acts as an anti-inflammatory agent in metabolic disorders yet an immune reinvigorator in the TME.

Vitamin D also acts on the gut microbiome (79): activity on intestinal epithelial cells alters commensal community composition (79), favoring expansion of Bacteroides fragilis, which positively regulates cancer immunity and augments responses to checkpoint blockade (79).

Coupled with these restored effector functions and microbiome alterations, differentiation into a highly resilient, central memory phenotype is directly facilitated by vitamin D. When baseline vitamin D reservoirs are sufficient, the TME may be remodeled such that the tumor becomes more sensitized to immune checkpoint blockade (80). Vitamin D deficiency is common across oncology populations, and in observational studies it has been associated with shorter OS and PFS (81, 82).

Iron: The metabolism of elemental iron introduces distinct physiological complexities. An intricate relationship connects systemic iron pools directly to immune cell functionality. Consequently, iron homeostasis acts as a critical determinant of the host immune response. A profound biological duality governs this micronutrient: adequate iron availability is required for T cells to undergo proliferation and execute effector functions. Both active DNA replication and sustained mitochondrial respiration rely critically on the availability of this metal (83). However, when iron accumulates excessively within the TME, cytotoxic cascades are initiated. This localized overload rapidly catalyzes the Fenton reaction, generating excessive quantities of reactive oxygen species (ROS). Ultimately, this oxidative stress triggers widespread ferroptosis, effectively compromising the functional integrity of the local immune landscape (84, 85).

However, the assumption that systemic ferroptosis inhibition synergizes with ICI therapy is a misconception, given a paradoxical crosstalk between iron-dependent ferroptosis and CD8+ T-cell function. Although effector CD8+ T cells drive tumor ferroptosis by secreting IFN-γ, which downregulates the system Xc- antiporter, these cells are themselves susceptible to ferroptotic stress in the TME (86): accumulating lipid peroxides and iron cause ferroptosis via CD36-dependent lipid uptake (87), impairing IFN-γ secretion and cytotoxicity and contributing to primary resistance (86, 87). This bottleneck limits broad systemic ferroptosis inducers (86), so shielding CD8+ T cells—by maintaining labile iron homeostasis or augmenting antioxidant capacity—is a key strategy in ICI-refractory tumors.

Conversely, inducing ferroptosis in tumor cells underlies part of the anti-tumor efficacy of ICIs: IFN-γ from reactivated CD8+ T cells downregulates the cystine-glutamate antiporter (SLC7A11) on malignant cells (88), disrupting their antioxidant defense and driving lethal ferroptosis.

The primary drivers of TME immunosuppression, Tregs and M2 macrophages, rely on glutathione peroxidase 4 (GPX4) to survive lipid peroxidation (89, 90). GPX4 inhibition induces ferroptosis in these populations, dismantling the immunosuppressive barrier and sensitizing tumors to ICI therapy.

Consequently, ideal clinical interventions must avoid broad, systemic modulation of ferroptosis. Instead, precise, cell-specific metabolic regulation is required. The primary therapeutic objective is to selectively target malignant populations and localized immunosuppressive lineages, particularly Tregs, for example through precision antibody-drug conjugates (ADCs) that enable selective depletion of these cells (91). Concurrently, a complementary objective is to protect effector CD8+ T cells from lipid peroxidation; targeted nanomedicine and metabolic-reprogramming strategies have been proposed toward this end, although selective protection of T cells remains to be demonstrated (92). Translating this mechanistic complexity into actionable clinical strategies is essential to overcome current bottlenecks in ICI resistance.

Zinc: As a cofactor for hundreds of enzymes, zinc underpins diverse immunological processes (93). Thymic development depends on adequate zinc, so its availability shapes the host’s T-cell repertoire, and mature immune function continues to rely on it. Zinc regulates T-cell signaling cascades and cytokine synthesis, supporting T-cell proliferation, differentiation, and activation (94).

Marked zinc deficiency induces generalized immune impairment, manifesting as T-cell dysfunction and dysregulated cytokines and increasing susceptibility to opportunistic infections (95–97). Zinc also modulates NF-κB signaling and metallothionein expression to preserve immune-cell integrity across innate and adaptive responses (94, 98). Within the TME, adequate zinc supports reversal of T-cell exhaustion and, by modulating the NF-κB network, exerts complementary anti-tumor and immunostimulatory effects (99).

#### 2.2.3 Emerging metabolic modulators: carbohydrates, amino acids, and retinoids

Beyond classical micronutrients, the concept of “nutritional interventions” has expanded to include monosaccharides, amino acid derivatives, and vitamin metabolites that act as epigenetic and transcriptional regulators of immune-cell fate (100).

Mannose: Cell-intrinsic mannose metabolism has emerged as a regulator of T-cell differentiation (101). Single-cell profiling shows diminished mannose metabolism is a hallmark of CD8+ T-cell exhaustion (101), whereas D-mannose supplementation restricts terminal exhaustion and preserves a stem-like phenotype (101). Mechanistically, D-mannose fuels O-GlcNAc transferase (OGT), which stabilizes β-catenin, sustaining Tcf7 expression and epigenetic stemness. Its capacity to maintain stemness may exceed that of interleukin 15 (IL-15), the current standard in adoptive cell therapy (ACT), offering a cost-effective route to ex vivo expansion of CAR-T cells and tumor-infiltrating lymphocytes (TILs) (101).

Taurine: Amino acid availability within the TME shapes the balance between activation and exhaustion, and taurine is a key contested molecule between tumor cells and CD8+ T cells (102). Tumor cells overexpress the transporter SLC6A6, outcompeting T cells for taurine (102)—an active mechanism of immune evasion: under chemotherapy pressure, gastric cancer upregulates the SP1-SLC6A6 axis to monopolize taurine (103). The resulting deficiency triggers endoplasmic reticulum (ER) stress and the PERK-JAK1-STAT3 cascade, upregulating ATF4, which transactivates checkpoint genes (PD-1, CTLA-4, TIM-3, TIGIT) and drives exhaustion. Taurine supplementation reverses this, relieving ER stress and reinvigorating exhausted CD8+ T cells to amplify checkpoint blockade (102).

Vitamin A and retinoid derivatives: The pleiotropic effects of Vitamin A, primarily mediated through retinoic acid (RA) and its receptors (RARs), profoundly reshape both the adaptive and innate immune compartments (104). Within the TME, RAR agonists (e.g., adapalene) execute a dual-faceted anti-tumor strategy. First, they act as potent pro-senescence agents, fundamentally reprogramming the tumor’s senescence-associated secretory phenotype (SASP). When combined with standard chemotherapies, RA signaling shifts the SASP from a tumor-promoting profile to a highly tumor-suppressive state, triggering robust intratumoral recruitment and activation of NK cells (104). Concurrently, RA directly rescues innate-like T cell populations from immune exhaustion. By repressing TIM-3 expression and promoting NF-κB activation, RA/RAR signaling effectively restores the potent cytotoxicity and cytokine production of γδ T cells in specific malignancies (105). These synergistic mechanisms suggest that targeted retinoid supplementation may help counteract TME-induced immunosuppression, although the supporting data are largely preclinical.

#### 2.2.4 The gut microbiome

Although not strictly a host nutrient, the intestinal microbiome is a critical conduit linking dietary intake to systemic immunity (106). The composition and metabolic output of these communities shape the host’s immune network; specific taxa ferment dietary fibers to produce short-chain fatty acids (SCFAs), most notably acetate, propionate, and butyrate (107).

Recent work has extended microbial metabolites beyond the mucosal barrier and Treg suppression (108, 109). Butyrate directly modulates cytotoxic CD8+ T-cell immunity (110): during chemotherapy it boosts CD8+ responses in an ID2-dependent manner via IL-12 signaling (110), and within checkpoint blockade it increases H3K27 acetylation at the Pdcd1 and Cd28 promoters in human CD8+ T cells (111), enhancing PD-1/CD28 expression and TCR signaling to amplify anti-PD-1 efficacy (111). Clinically, elevated serum butyrate correlates with response to oxaliplatin-based chemotherapy or anti-PD-1 immunotherapy (110, 111).

The microbiome’s ecological balance also governs irAE risk (112, 113). Alterations in gut microbiota composition and function are linked to irAE pathogenesis, particularly ICI-induced colitis (114), and distinct microbial signatures both shape the inflammatory milieu and hold potential as predictive biomarkers for these toxicities (114).

These dynamics point to a promising therapeutic frontier (115): deliberate microbiome modulation may simultaneously enhance efficacy and mitigate severe irAEs (114, 115). Strategies under clinical evaluation include fecal microbiota transplantation (FMT), probiotics, prebiotics, postbiotics, and dietary interventions (114, 115). FMT, for instance, has restored therapeutic sensitivity in patients previously refractory to checkpoint blockade (116).

In summary, the host’s microbiome, endocrine status, and nutritional foundation form an interconnected network that shapes every phase of the anti-tumor immune cycle, from the ontogeny and maturation of immune effectors to their cytotoxic execution within the TME.

Together, these systemic variables establish a defining host macroenvironment whose integrity is a key determinant of immunotherapeutic efficacy. Elucidating the synergy among these host-level factors provides a mechanistic foundation for precision oncology and is essential to maximize response rates and improve long-term outcomes.

## 3 Clinical challenges and opportunities

Integrating host endocrine and nutritional biomarkers into ICI management presents notable challenges alongside distinct opportunities, and calls for a comprehensive, systemic approach to patient assessment and management (**Table 1**).

**Table 1:** Host endocrine, nutritional and metabolic biomarkers relevant to ICI therapy: biological rationale, current clinical evidence, prognostic versus predictive role, major confounders, and readiness for clinical implementation.

Host factor / biomarker	Biological rationale	Available clinical evidence	Prognostic vs predictive	Major confounders	Readiness for implementation
Baseline cortisol / HPA-axis activation	GC-mediated suppression of T-cell activation, DC maturation and NK function; expansion of Tregs/MDSCs (largely preclinical)	Observational cohorts across melanoma, NSCLC, RCC and haematological malignancies link high baseline cortisol to lower ORR and shorter PFS/OS	Prognostic; predictive role not established	Advanced disease, exogenous glucocorticoids, acute stress, assay/timing variability	Low – no standardized threshold or validated intervention
Baseline (pre-treatment) hypothyroidism	Thyroid hormone supports antigen presentation and T-cell effector metabolism (preclinical)	Cohort studies associate baseline hypothyroidism with poorer outcomes	Prognostic	Overall metabolic/performance status, comorbidity, medication	Low-moderate – routinely measured but not validated as decision tool
Treatment-emergent thyroid dysfunction (thyroiditis / thyrotoxicosis)	Reflects on-target systemic immune activation	Meta-analyses report longer OS/PFS in patients developing ICI-related thyroid dysfunction	Apparent predictive/pharmacodynamic marker, but confounded	Immortal-time bias, treatment duration, surveillance intensity	Low – requires time-dependent prospective validation
Insulin resistance / hyperinsulinaemia	Impaired T-cell metabolic fitness; myeloid-driven suppression (preclinical + observational)	Cohorts associate pre-treatment insulin resistance with inferior outcomes	Prognostic	Obesity, diabetes, concomitant metabolic therapy	Low
Body composition / BMI (obesity paradox)	Adipose-derived signalling and reserve may modulate immunity and tolerance	Retrospective cohorts/pooled analyses associate higher BMI with better outcomes in melanoma/NSCLC	Prognostic; likely confounded	Collider/selection bias, reverse causation, sarcopenic obesity, smoking	Low for BMI; CT-based body composition more informative
Serum albumin / protein-energy malnutrition	Substrate limitation impairs lymphocyte proliferation and effector-protein synthesis	Multiple cohorts/meta-analyses link hypoalbuminaemia to shorter PFS/OS	Prognostic	Systemic inflammation, hepatic/renal status, hydration	Moderate – inexpensive and widely available
Composite indices (PNI, PINI, GPS)	Combine nutritional (albumin) and inflammatory/immune (lymphocyte or monocyte) components	Retrospective/validation cohorts (NSCLC, GI) associate low PNI / abnormal PINI with worse OS and primary resistance	Prognostic	No standardized cut-offs, cohort heterogeneity	Moderate – promising but needs threshold standardization
Vitamin D	Context-dependent, pleiotropic immunomodulation via VDR (mostly preclinical)	Observational data link deficiency to shorter OS/PFS; no positive interventional RCT	Prognostic (association); predictive/interventional benefit unproven	Season, supplementation, inflammation, comorbidity	Low – supplementation benefit not validated
Iron metabolism	Dual role: required for T-cell function yet drives ferroptosis of CD8+ T cells in the TME (preclinical)	Predominantly preclinical/mechanistic	Not established	Inflammation-driven hepcidin, anaemia of malignancy	Very low – conceptual
Gut microbiome	SCFA/butyrate-mediated modulation of CD8+ T-cell immunity and irAE risk	Observational associations; early FMT and microbiome-modulation trials	Emerging predictive candidate	Diet, antibiotics, geography, methodology	Low-moderate – active clinical evaluation
GLP-1 receptor agonists (intervention)	Reduce insulin resistance and low-grade inflammation; possible microbiome/immune effects (hypothesized)	No prospective ICI-combination data; oncological signals are epidemiological/preventive	Speculative therapeutic hypothesis	Sarcopenia risk, cachexia, selection bias	Very low – requires stratified prospective RCTs

BMI, body mass index; DC, dendritic cell; FMT, faecal microbiota transplantation; GC, glucocorticoid; GI, gastrointestinal; GLP-1, glucagon-like peptide-1; GPS, Glasgow Prognostic Score; HPA, hypothalamic–pituitary–adrenal; ICI, immune checkpoint inhibitor; irAE, immune-related adverse event; MDSC, myeloid-derived suppressor cell; NK, natural killer; NSCLC, non-small cell lung cancer; ORR, objective response rate; OS, overall survival; PFS, progression-free survival; PINI, Prognostic Immune and Nutritional Index; PNI, Prognostic Nutritional Index; RCC, renal cell carcinoma; RCT, randomized controlled trial; SCFA, short-chain fatty acid; TME, tumour microenvironment; Treg, regulatory T cell; VDR, vitamin D receptor.

### 3.1 Practical hurdles in the clinic

#### 3.1.1 Bidirectional complexity of endocrine-immune interactions

Host hormonal-nutritional status and the immune system interact within complex, bidirectional feedback loops rather than linear pathways. This is compounded by ICI-induced endocrinopathies, including hypophysitis, thyroiditis, adrenalitis, and type 1 diabetes (117), whose management demands multidisciplinary collaboration many practices struggle to sustain. The dynamic also obscures causality: it remains debated whether pre-treatment endocrine dysregulation drives poor outcomes or reflects advanced disease, and whether treatment-emergent toxicity indicates immune activation or collateral damage—ambiguities requiring prospective investigation.

#### 3.1.2 Standardization deficits and threshold variability

The field lacks consensus guidelines defining optimal biomarker thresholds for ICI efficacy, so groups adopt arbitrary cutoffs. Definitive thresholds for the Prognostic Immune and Nutritional Index (PINI), serum Vitamin D targets, and cutoffs for overt thyrotoxicosis remain unestablished, limiting application and cross-study comparability (118, 119). Baseline timing is also undefined—whether it should reflect the host one week or one month before ICI initiation; standardizing this window is a prerequisite for multi-center trials, as is stringent standardization of pre-analytical and analytical protocols (sample timing, storage, reagents).

#### 3.1.3 Mitigating the impact of confounding variables

The physiological baseline of an oncology patient is inherently heterogeneous, shaped by tumor histology, disease burden, demographics such as age and sex, comorbidities such as diabetes, and effects of prior chemotherapy and radiation (120). Concurrent pharmacotherapy adds interference—GCs, for instance, alter the profiles under evaluation. Validating these biomarkers therefore requires multivariate modeling to adjust for confounders; without it, claims of causality remain compromised.

#### 3.1.4 Optimizing intervention strategies and timing

While identifying a prognostic biomarker is important, establishing the appropriate countermeasure is a separate challenge, and optimal intervention protocols remain undefined. The value of correcting vitamin D deficiency to enhance responses, or of pharmacologically suppressing baseline cortisol before treatment, is unproven. Any prospective intervention requires randomized controlled trials to confirm safety and ensure benefit outweighs the risks of adverse events and patient burden (121, 122).

### 3.2 Translational opportunities

Despite these challenges, integrating host factors into clinical decision-making offers a real opportunity to optimize ICI therapy and move oncology toward a more precise, individualized paradigm.

#### 3.2.1 Composite prognostic indices and systemic immune readiness assessment systems

In translating fragmented endocrine and nutritional biomarkers into actionable clinical tools, single indicators often lack sufficient predictive power. Therefore, it is imperative to investigate and apply composite prognostic scoring systems, such as the PNI (Prognostic Nutritional Index), the Prognostic Immune and Nutritional Index (PINI), and the GPS (Glasgow Prognostic Score) (123, 124). The PINI, for instance, provides a multidimensional assessment by integrating serum albumin levels, which reflect the host’s protein anabolic status, with absolute monocyte counts, a proxy for systemic inflammatory burden.

Recent retrospective cohort studies and multicenter validation models indicate that in patients with NSCLC and gastrointestinal malignancies, a low pre-treatment PNI or an abnormal PINI score serves as an independent negative prognostic factor for OS and strongly correlates with primary resistance to ICIs (124, 125). The prognostic value of these integrated scoring systems stems from their ability to capture dysregulation within key immunometabolic pathways at a macro-scale. For example, T cells utilize the transmembrane transporter SLC7A5 to directly sense nutrient deprivation resulting from the depletion of essential amino acids, specifically leucine. This sensing mechanism inhibits intracellular mTORC1 signaling, ultimately driving T cells into a state of metabolic failure and functional exhaustion (126–128).

We therefore advocate including a composite Host Health Index, incorporating nutritional and endocrine parameters, alongside genomic biomarkers such as TMB in future Molecular Tumor Board deliberations—a shift from evaluating tumor immunogenicity alone toward quantifying the host’s metabolic capacity to sustain an anti-tumor immune response.

#### 3.2.2 Strategic clinical interventions: the dual nature of glucagon-like peptide-1 receptor agonists

Identifying deleterious host factors provides critical opportunities for proactive interventions designed to optimize the systemic milieu. Within this therapeutic landscape, the potential clinical application of GLP-1RAs, notably semaglutide, has attracted growing interest in immuno-oncology. Originally developed for type 2 diabetes and obesity, GLP-1RAs have recently been associated with potential oncological benefits in observational data, although these findings are hypothesis-generating. For instance, large-scale real-world cohort data presented at the 2026 ASCO Gastrointestinal Cancers Symposium confirmed that GLP-1RA usage is associated with a 36% reduction in colorectal cancer incidence. Notably, this effect remains consistent regardless of baseline body mass index (BMI) or glycemic status (129). While this epidemiological milestone firmly establishes the preventive value of GLP-1RAs, driven largely by the amelioration of systemic metabolic load and the suppression of PI3K/Akt/mTOR proliferative signaling to halt early oncogenesis, it strictly reflects primary cancer prevention.

Preventing malignant transformation in normal cells relies on a fundamentally distinct biological logic compared to reversing the profound exhaustion of CD8+ T cells within the highly hostile and immunosuppressive microenvironment of established advanced tumors. Therefore, to rigorously justify the integration of GLP-1RAs with ICI therapy, the mechanistic focus must pivot from tumor prevention to the reversal of host immunometabolic exhaustion. In the context of advanced malignancies, chronic hyperinsulinemia imposes continuous functional suppression on effector T cells. By improving insulin resistance, attenuating systemic low-grade inflammation, and potentially enhancing CD8+ T-cell anti-tumor activity via gut microbiota remodeling, GLP-1RAs might alleviate part of this suppressive burden and provide immune cells with a more favorable metabolic environment. It has also been hypothesized that their immunomodulatory properties could offer organ-specific protection against autoimmune-mediated toxicities such as ICI-induced hypophysitis and diabetes; however, this protective effect has not been demonstrated in prospective clinical studies (117). This putative dual action, improving systemic metabolic fitness while potentially mitigating irAEs, has been proposed as a conceptual route toward efficacy-toxicity decoupling; at present, however, this concept is speculative and lacks direct prospective clinical evidence.

However, integrating these agents into clinical oncology introduces a profound logical conflict, particularly concerning the well-documented obesity paradox. While a preserved adipose reservoir may confer metabolic resilience during ICI therapy, the potent anorexigenic and weight-loss effects of GLP-1RAs pose severe risks. Blindly administering these agents could inadvertently precipitate PEM, cachexia, and further declines in serum albumin levels, which serves as a critical systemic indicator of ICI resistance (123). It is important to note, however, that current evidence regarding the exact risk of GLP-1RA-induced sarcopenia remains inconsistent (130). While body composition substudies of major randomized controlled trials demonstrate that GLP-1RA-induced weight loss is predominantly driven by fat mass reduction, absolute lean mass frequently decreases in tandem, even though relative lean mass percentages may be preserved (131–133). Conversely, real-world observational data and pharmacovigilance signals suggest that vulnerable subgroups, such as the elderly or patients with tumor-induced cachexia, low physical activity, and inadequate protein intake, face a substantially elevated risk of critical muscle and functional strength loss (134). These discrepancies likely stem from brief follow-up durations, heterogeneous definitions of sarcopenia, and varying measurement modalities (e.g., DXA versus CT) across different studies (130, 134).As demonstrated by Chaunzwa et al. in a large-scale 2024 JAMA Oncology observational cohort of 1,791 patients with NSCLC, progressive skeletal muscle depletion (sarcopenia) during systemic therapy, quantified via AI-driven deep learning of CT scans, is associated with abbreviated survival and serves as a marker of poor oncologic outcomes (15). To elevate this clinical observation into a mechanistic framework, we hypothesize that skeletal muscle may function far beyond mere mechanical locomotion, potentially operating as a highly active endocrine organ (135). The preservation of skeletal muscle mass might be immunologically critical, as it conceptually serves as a putative reservoir for myokines, most notably interleukin-15 (IL-15) and specific immunomodulatory isoforms of interleukin-6 (IL-6) (135). These muscle-derived cytokines are postulated to be important for maintaining the homeostatic survival, basal proliferation, and longevity of peripheral memory CD8+ T cell pools (136). Consequently, we hypothesize that the rapid onset of GLP-1RA-induced sarcopenia could represent more than structural frailty; it may compromise this putative “cytokine reservoir”, posing a theoretical risk to the host’s long-term anti-tumor immunological memory (137, 138).

To resolve this conflict, clear boundary conditions must govern the oncological use of GLP-1RAs. The primary objective of introducing GLP-1RAs in immuno-oncology is not weight reduction; it is to reverse systemic low-grade inflammation, address insulin resistance, and reconstruct the intestinal microecology. We therefore advocate for a phenotype-specific model: GLP-1RAs should be restricted to patients with severe metabolic syndrome or pronounced visceral adiposity.

Furthermore, to safely unlock the immunotherapeutic potential of GLP-1RAs, a mandatory combinatorial strategy is required. Future clinical evaluations must prioritize stratified, prospective RCTs with a minimum follow-up of 12 to 24 months (134). The deployment of these metabolic modulators must be inextricably coupled with precision clinical nutritional support, specifically high-protein dietary regimens, and tailored resistance exercise prescriptions to counteract potential treatment-induced muscle wasting (134). Alongside core oncological endpoints, these future studies should routinely integrate comprehensive functional and compositional metrics, including continuous CT-derived absolute muscle mass quantification, grip strength, and the Short Physical Performance Battery (SPPB) (15, 134). To translate this into actionable clinical governance, we strongly advocate that multidisciplinary Molecular Tumor Boards (MTBs) establish a strict, individualized safe lower limit for the Skeletal Muscle Index (SMI) prior to treatment (15). Should a patient’s SMI breach this critical threshold during co-administration, GLP-1RA interventions must be immediately suspended to prevent the irreversible collapse of the host’s immunological infrastructure.

Looking ahead, the next step in endocrine-oncology will require therapeutic strategies that achieve genuine “muscle-preserving fat reduction.” Next-generation agents, such as multi-receptor incretin agonists (e.g., GLP-1/GIP/glucagon tri-agonists), or the combination of metabolic modulators with targeted myostatin (GDF-8) inhibitors, represent a promising frontier. By uncoupling visceral adiposity depletion from skeletal muscle wasting, such regimens could provide the immunometabolic substrate needed to safely and sustainably maximize ICI efficacy.

## 4 Future perspectives

The integration of hormonal and nutritional biomarkers holds substantial potential to guide individualized immunotherapy. To realize it, future research must move beyond validating existing markers toward interdisciplinary expansion across several frontier domains.

First, static single-timepoint biomarkers cannot capture the dynamic host-tumor interaction. Future work should integrate artificial intelligence (AI) to process longitudinal, non-linear multimodal data, combining macro-physiological trajectories from electronic health records (EHRs)—weight, glycemic, and cortisol histories—with single-cell profiling and spatial transcriptomics of the TME. Machine-learning synthesis could yield Dynamic Response Network Models that track the host’s metabolic foundation and forecast both ICI efficacy and toxicity (12).

Second, future research must establish reproducible, standardized detection protocols. Through prospective multi-center cohorts and reference-laboratory collaborations, it is important to define interpretable cut-off values and uniform sampling windows for pre- and peri-treatment assessments (139), and to standardize pre-analytical workflows—sample selection, collection, centrifugation, cryopreservation—with external quality assessment (EQA), calibrators, and inter-assay control (140, 141). This framework should enter regulatory evaluation and clinical guidelines early (142), balancing accessibility, turnaround, and cost.

Third, a key frontier combines metabolic or nutritional modulators with ICIs to optimize the host immunometabolic substrate and sensitize the tumor. Aberrant tumor metabolism, for instance, saturates the TME with inhibitory metabolites such as lactate; pairing ICIs with agents like lactate efflux inhibitors can dismantle these barriers and enhance CD8+ T-cell infiltration and cytolytic function (143).

Expanding the immunonutritional toolkit to emerging metabolic modulators is a promising frontier (100): D-mannose can preserve epigenetic stemness via β-catenin O-GlcNAcylation (101), taurine supplementation can override ER-stress-induced checkpoint transactivation (102), and retinoic acid receptor (RAR) agonists can reprogram senescence-associated secretory phenotypes to enhance NK-cell recruitment (104). These act not merely as supportive care but as agents capable of uncoupling immune expansion from terminal exhaustion (100).

The translation of classical micronutrients like vitamin D must account for their context-dependent pleiotropy (75). Active vitamin D analogs could be deployed with ICIs to epigenetically silence checkpoints (e.g., PD-1, TIM-3) and reverse T-cell exhaustion (78) while ensuring the PLC-γ1 upregulation required for naive T-cell priming (77). However, their capacity to suppress inflammatory cytokines via FBP1-mediated glycolysis inhibition under distinct conditions (75) means precision dosing and stratification by host metabolic profile will be essential.

At the systemic level, GLP-1RAs may modulate chronic inflammation and address insulin resistance; their integration with ICIs and nutritional support warrants prospective study (117). Another frontier is neutralizing HPA-axis immunosuppression: given the Tsc22d3 and ACBP/DBI axis in disrupting type I interferon signaling in murine models, ACBP/DBI-targeted antibodies merit investigation as adjuvant immunotherapy. Though speculative, preclinical data suggest neutralizing ACBP/DBI could counteract the tolerance induced by iatrogenic glucocorticoids or tumor-driven hypercortisolemia (34), restoring dendritic-cell function under stress. Gut-microbiome interventions—probiotics, prebiotics, FMT, or modulators like vitamin D to expand commensals such as Bacteroides fragilis (79)—offer a further auxiliary strategy.

Finally, the hypotheses and models discussed here require validation through rigorously designed prospective trials that move beyond single-dimensional assessment. Rather than using host biomarkers solely for stratification, trials should incorporate active intervention arms—comparing ICI therapy plus standardized nutrition and exercise against ICI therapy with conventional care—to determine whether active metabolic remodeling improves long-term survival. This will require collaboration among oncology, endocrinology, clinical nutrition, computational biology, and immunology.

## 5 Conclusion

ICIs represent a major advance, yet the heterogeneity of clinical responses calls for a paradigm shift beyond the tumor-centric perspective. This review has delineated the central role of host endocrine and nutritional status in orchestrating anti-tumor immunity and shaping outcomes. Our objective extends beyond identifying novel biomarkers; we advocate a broader change in the conceptual framework of cancer immunotherapy.

**Figure 1:**
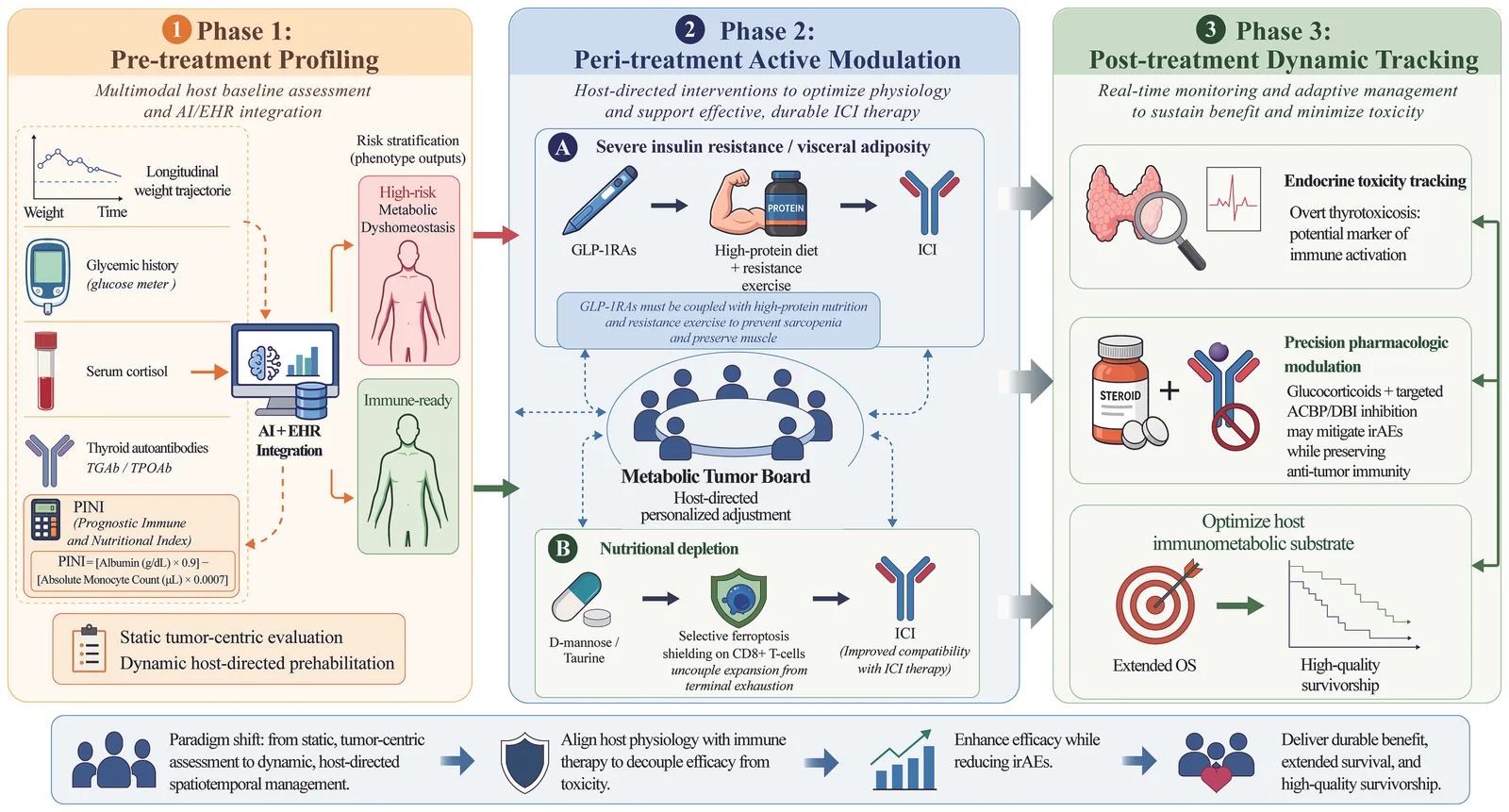
Spatiotemporal clinical management blueprint for achieving efficacy-toxicity decoupling during immune checkpoint inhibitor therapy. This conceptual framework illustrates a proposed transition from static, tumor-centric stratification toward dynamic, host-directed assessment, prehabilitation, and longitudinal management across three interconnected clinical phases. Importantly, specific evidence-based quantitative thresholds, such as definitive PINI cutoffs for phenotype stratification, standardized serum Vitamin D targets, and exact diagnostic parameters defining overt thyrotoxicosis, are currently lacking. Consequently, these parameters are presented as part of a theoretical model and require rigorous definition and validation in future prospective clinical trials. Phase 1: Pre-treatment profiling. Before initiation of immune checkpoint inhibitor (ICI) therapy, a comprehensive host-level assessment is performed to characterize metabolic, endocrine, immune, and nutritional readiness. Longitudinal weight trajectories, glycemic history, serum cortisol levels, thyroid autoantibodies, and the Prognostic Immune and Nutritional Index (PINI) are integrated with clinical information derived from electronic health records (EHRs). In this framework, PINI is calculated as ([Albumin (g/dL) × 0.9] − [Absolute Monocyte Count (/μL) × 0.0007]). Artificial intelligence-assisted multimodal integration may facilitate phenotype-based risk stratification, distinguishing patients with high-risk metabolic dyshomeostasis, such as severe insulin resistance or visceral adiposity, from those with a comparatively immune-ready physiological profile. The output of this phase is an individualized prehabilitation and monitoring plan rather than a single static biomarker classification. Phase 2: Peri-treatment active modulation. Host-directed interventions are selected and dynamically adjusted through multidisciplinary review by a Metabolic Tumor Board. In patients with severe insulin resistance or visceral adiposity (Path A), glucagon-like peptide-1 receptor agonists (GLP-1RAs) may be explored as metabolic adjuncts to ICI therapy. Because these agents may induce weight loss and skeletal muscle depletion, their use should be accompanied by high-protein nutritional support, resistance exercise, and serial body-composition monitoring to preserve muscle mass. In patients with nutritional depletion or impaired immunometabolic fitness (Path B), correction of clinically documented deficiencies and investigation of metabolic modulators, including D-mannose and taurine, may support immune-cell function. Cell-selective ferroptosis modulation is presented as an investigational strategy intended to protect effector CD8^+^ T cells from lipid peroxidation while avoiding nonspecific systemic inhibition of tumor-cell ferroptosis. These peri-treatment interventions are proposed to improve host compatibility with ICI therapy and require validation in prospective clinical trials. Phase 3: Post-treatment dynamic tracking. Endocrine function, nutritional status, body composition, immune-related adverse events (irAEs), treatment response, and survival outcomes are monitored longitudinally. The development of ICI-associated thyroid dysfunction, particularly overt thyrotoxicosis, may represent a dynamic clinical feature associated with systemic immune activation, but it should not be interpreted as a validated surrogate endpoint. irAEs should be detected early, graded systematically, and managed according to established clinical guidance. Glucocorticoids remain standard treatment for selected moderate-to-severe irAEs, whereas targeted modulation of the acyl-CoA-binding protein/diazepam-binding inhibitor (ACBP/DBI) axis is shown only as a proposed investigational approach that might reduce glucocorticoid-related immune suppression without compromising antitumor activity. Continuous feedback from this phase informs subsequent treatment adjustment and survivorship care. Overall, the three phases form an iterative management cycle designed to optimize the host immunometabolic substrate, preserve immune competence, reduce clinically significant toxicity, and potentially improve durable disease control, overall survival, and quality of survivorship. ACBP/DBI, acyl-CoA-binding protein/diazepam-binding inhibitor; AI, artificial intelligence; EHR, electronic health record; GLP-1RA, glucagon-like peptide-1 receptor agonist; ICI, immune checkpoint inhibitor; irAE, immune-related adverse event; OS, overall survival; PINI, Prognostic Immune and Nutritional Index; TGAb, thyroglobulin antibody; TPOAb, thyroid peroxidase antibody. Figure 1 was conceptually designed and manually prepared by the authors using Adobe Illustrator 2024. No generative AI or AI-assisted image-generation tool was used in its creation, modification, or enhancement. The original editable source file is available upon request.

We propose that future immuno-oncology must transition from static stratification toward proactive prehabilitation. Host hormonal and nutritional parameters should no longer be relegated to the status of passive prognostic indicators. Instead, they must be recognized as actionable therapeutic targets. As explored herein, the integration of metabolic modulators, such as GLP-1RAs, with precision clinical nutritional support represents a hypothesis-generating strategy toward efficacy-toxicity decoupling that requires prospective validation. To translate this vision into clinical reality, we propose a spatiotemporal management blueprint spanning pre-treatment profiling, active peri-treatment modulation, and dynamic post-treatment tracking (**Figure 1**). This approach attenuates systemic low-grade inflammation and sensitizes the tumor to immunotherapy while concurrently fortifying the host against the risks of malnutrition and cachexia.

Translating this vision into practice requires collaboration beyond traditional oncology, integrating immuno-oncology, endocrinology, clinical nutrition, and computational biology. We advocate institutionalizing Molecular and Metabolic Tumor Boards within standard care. Elevating active remodeling of the host’s metabolic-endocrine network to parity with genomic targeting is essential to realize precision survivorship medicine and secure durable benefit and quality of life for a growing patient population.

## Data Availability

The original contributions presented in the study are included in the article/supplementary material. Further inquiries can be directed to the corresponding authors.

## References

[1] GBD 2023 Cancer Collaborators . The global, regional, and national burden of cancer, 1990-2023, with forecasts to 2050: a systematic analysis for the Global Burden of Disease Study 2023. Lancet. (2025) 406:1565–86. doi: 10.1016/S0140-6736(25)01635-6 PMC12687902 41015051

[2] Robert C . A decade of immune-checkpoint inhibitors in cancer therapy. Nat Commun. (2020) 11:3801. doi: 10.1038/s41467-020-17670-y 32732879 PMC7393098

[3] Lee JB Kim HR Ha SJ . Immune checkpoint inhibitors in 10 years: Contribution of basic research and clinical application in cancer immunotherapy. Immune Netw. (2022) 22:e2. doi: 10.4110/in.2022.22.e2 35291660 PMC8901707

[4] Petrelli F Ghidini A Parati MC Borgonovo K Rossitto M Ghilardi M . Immune checkpoint inhibitors beyond progression in various solid tumors: A systematic review and pooled analysis. J Clin Med. (2025) 14:6680. doi: 10.3390/jcm14186680 41010884 PMC12470277

[5] Tarhini AA Joshi I Garner F . Sargramostim and immune checkpoint inhibitors: combinatorial therapeutic studies in metastatic melanoma. Immunotherapy. (2021) 13:1011–29. doi: 10.2217/imt-2021-0119 34157863

[6] Shiravand Y Khodadadi F Kashani SMA Hosseini-Fard SR Hosseini S Sadeghirad H . Immune checkpoint inhibitors in cancer therapy. Curr Oncol. (2022) 29:3044–60. doi: 10.3390/curroncol29050247 35621637 PMC9139602

[7] Doroshow DB Bhalla S Beasley MB Sholl LM Kerr KM Gnjatic S . PD-L1 as a biomarker of response to immune-checkpoint inhibitors. Nat Rev Clin Oncol. (2021) 18:345–62. doi: 10.1038/s41571-021-00473-5 33580222

[8] Alsaafeen BH Ali BR Elkord E . Resistance mechanisms to immune checkpoint inhibitors: updated insights. Mol Cancer. (2025) 24:20. doi: 10.1186/s12943-024-02212-7 39815294 PMC11734352

[9] Wang DY Salem JE Cohen JV Chandra S Menzer C Ye F . Fatal toxic effects associated with immune checkpoint inhibitors: A systematic review and meta-analysis. JAMA Oncol. (2018) 4:1721–8. doi: 10.1001/jamaoncol.2018.3923 30242316 PMC6440712

[10] Huang RSP Haberberger J Severson E Duncan DL Hemmerich A Edgerly C . A pan-cancer analysis of PD-L1 immunohistochemistry and gene amplification, tumor mutation burden and microsatellite instability in 48,782 cases. Mod Pathol. (2021) 34:252–63. doi: 10.1038/s41379-020-00664-y 32884129

[11] Chalmers ZR Connelly CF Fabrizio D Gay L Ali SM Ennis R . Analysis of 100,000 human cancer genomes reveals the landscape of tumor mutational burden. Genome Med. (2017) 9:34. doi: 10.1186/s13073-017-0424-2 28420421 PMC5395719

[12] Bai R Lv Z Xu D Cui J . Predictive biomarkers for cancer immunotherapy with immune checkpoint inhibitors. biomark Res. (2020) 8:34. doi: 10.1186/s40364-020-00209-0 32864131 PMC7450548

[13] Huang N Guan Y . Pre-treatment endocrine-nutritional signatures predict clinical benefit from PD-1/PD-L1 blockade in hematologic Malignancies. Front Nutr. (2026) 12:1753660. doi: 10.3389/fnut.2025.1753660 41726000 PMC12921361

[14] Zeng Y Hu CH Li YZ Zhou JS Wang SX Liu MD . Association between pretreatment emotional distress and immune checkpoint inhibitor response in non-small-cell lung cancer. Nat Med. (2024) 30:1680–8. doi: 10.1038/s41591-024-02929-4 38740994 PMC11186781

[15] Chaunzwa TL Qian JM Li Q Ricciuti B Nuernberg L Johnson JW . Body composition in advanced non-small cell lung cancer treated with immunotherapy. JAMA Oncol. (2024) 10:773–83. doi: 10.1001/jamaoncol.2024.1120 38780929 PMC11117154

[16] Schaubaecher JB Smiljanov B Haring F Steiger K Wu Z Ugurluoglu A . Procoagulant platelets promote immune evasion in triple-negative breast cancer. Blood. (2024) 144:216–26. doi: 10.1182/blood.2023022928 38648571

[17] Zheng JY Zhu J Wang Y Tian ZZ . Effects of acupuncture on hypothalamic-pituitary-adrenal axis: Current status and future perspectives. J Integr Med. (2024) 22:445–58. doi: 10.1016/j.joim.2024.06.004 38955651

[18] Ruiz-Pozo VA Cadena-Ullauri S Tamayo-Trujillo R Guevara-Ramirez P Paz-Cruz E Castaneda Catana MA . Interplay between endogenous hormones and immune systems in human metapneumovirus pathogenesis and management. Front Pharmacol. (2025) 16:1568828. doi: 10.3389/fphar.2025.1568828 40176892 PMC11961889

[19] Schrepf A Clevenger L Christensen D DeGeest K Bender D Ahmed A . Cortisol and inflammatory processes in ovarian cancer patients following primary treatment: relationships with depression, fatigue, and disability. Brain Behav Immun. (2013) 30 Suppl:S126–134. doi: 10.1016/j.bbi.2012.07.022 22884960 PMC3697797

[20] Phillips KM Antoni MH Lechner SC Blomberg BB Llabre MM Avisar E . Stress management intervention reduces serum cortisol and increases relaxation during treatment for nonmetastatic breast cancer. Psychosom Med. (2008) 70:1044–9. doi: 10.1097/psy.0b013e318186fb27 18842742 PMC5761725

[21] Merk VM Grob L Fleischmann A Brunner T . Human lung carcinomas synthesize immunoregulatory glucocorticoids. Genes Immun. (2023) 24:52–6. doi: 10.1038/s41435-023-00194-y 36653475 PMC9935384

[22] Swatler J Ju YJ Anderson AC Lugli E . Tumors recycle glucocorticoids to drive Treg-mediated immunosuppression. J Clin Invest. (2023) 133:e173141. doi: 10.1172/jci173141 37712416 PMC10503790

[23] Aggrey-Fynn JE Manjunath M Rajput A Abdelrahman AM Thiel J Truty MJ . Therapeutic targeting of FOSL1 and RELA-dependent transcriptional mechanisms to suppress pancreatic cancer metastasis. Cell Death Dis. (2025) 16:504. doi: 10.1038/s41419-025-07810-x 40634284 PMC12241458

[24] Huang H Liao H Chen Y Hu M Jiang X Yu Q . Disruption of the Clock Component BMAL1 in HDM-induced asthma causes GC resistance in airway epithelial cells by regulating GR phosphorylation through the DUSP4-p38MAPK pathway. Int J Biol Sci. (2025) 21:6482–500. doi: 10.7150/ijbs.119486 41208877 PMC12594601

[25] Kobayashi K Inohara N Hernandez LD Galan JE Nunez G Janeway CA . RICK/Rip2/CARDIAK mediates signalling for receptors of the innate and adaptive immune systems. Nature. (2002) 416:194–9. doi: 10.1038/416194a 11894098

[26] Van Laethem F Baus E Smyth LA Andris F Bex F Urbain J . Glucocorticoids attenuate T cell receptor signaling. J Exp Med. (2001) 193:803–14. doi: 10.1084/jem.193.7.803 11283153 PMC2193373

[27] Luu TV Fleischer Hach L Seremet T Leuchte K Thor Straten P Holmen Olofsson G . Exercise duration modulates cortisol release and chronic cortisol exposure jeopardises T cell effector functions. Immunology. (2026) 177:94–105. doi: 10.1111/imm.70028 40796353 PMC12665805

[28] Brown JR Pedersen B Long GV Maher NG Lim SY Rizos H . Prednisolone modulates CD8(+) and regulatory T-cell activity to dampen response to immune checkpoint inhibitor therapy in melanoma. Oncoimmunology. (2026) 15:2643494. doi: 10.1080/2162402x.2026.2643494 41807927 PMC12987513

[29] Collins CP Khuat LT Sckisel GD Vick LV Minnar CM Dunai C . Systemic immunostimulation induces glucocorticoid-mediated thymic involution succeeded by rebound hyperplasia which is impaired in aged recipients. Front Immunol. (2024) 15:1429912. doi: 10.3389/fimmu.2024.1429912 39315105 PMC11416920

[30] Gertel S Polachek A Furer V Dovrat TO Avaky C Broyde A . The mitigating effect of combined glucocorticoids with immune checkpoint inhibitors on lymphocyte activation gene-3 and programmed death-1 expression. Eur J Immunol. (2025) 55:e70033. doi: 10.1002/eji.70033 40788332 PMC12338123

[31] He XY Gao Y Ng D Michalopoulou E George S Adrover JM . Chronic stress increases metastasis via neutrophil-mediated changes to the microenvironment. Cancer Cell. (2024) 42:474–86:e412. doi: 10.1016/j.ccell.2024.01.013 38402610 PMC11300849

[32] Piemonti L Monti P Allavena P Sironi M Soldini L Leone BE . Glucocorticoids affect human dendritic cell differentiation and maturation. J Immunol. (1999) 162:6473–81. doi: 10.4049/jimmunol.162.11.6473 10352262

[33] Rea D van Kooten C van Meijgaarden KE Ottenhoff TH Melief CJ Offringa R . Glucocorticoids transform CD40-triggering of dendritic cells into an alternative activation pathway resulting in antigen-presenting cells that secrete IL-10. Blood. (2000) 95:3162–7. doi: 10.1182/blood.v95.10.3162 10807783

[34] Pan H Shen Z Zhao L Liu P Jin Z Piard E . Neutralization of acyl-CoA-binding protein attenuates glucocorticoid-mediated suppression of cancer immunosurveillance. Proc Natl Acad Sci USA. (2026) 123:e2518983123. doi: 10.1073/pnas.2518983123 41779791 PMC12974490

[35] Trojandt S Bellinghausen I Reske-Kunz AB Bros M . Tumor-derived immuno-modulators induce overlapping pro-tolerogenic gene expression signatures in human dendritic cells. Hum Immunol. (2016) 77:1223–31. doi: 10.1016/j.humimm.2016.08.014 27593566

[36] Taves MD Otsuka S Taylor MA Donahue KM Meyer TJ Cam MC . Tumors produce glucocorticoids by metabolite recycling, not synthesis, and activate Tregs to promote growth. J Clin Invest. (2023) 133:e164599. doi: 10.1172/jci164599 37471141 PMC10503810

[37] Taya M Gray E Srivastava T Chee WY Fleming GF DeVilbiss AW . Ovarian cancer cell glucocorticoid receptor activation increases myeloid-derived suppressor cell tumor infiltration. Endocrinology. (2026) 167:bqag019. doi: 10.1210/endocr/bqag019 41732082

[38] Kaliyamoorthi K . Steroid-induced modulation of the tumor immune microenvironment: Implications for cancer progression and immunotherapy outcomes. J Steroid Biochem Mol Biol. (2026) 259:106978. doi: 10.1016/j.jsbmb.2026.106978 41775287

[39] Karmakar S Chatterjee M Basu M Ghosh MK . CK2: The master regulator in tumor immune-microenvironment - A crucial target in oncotherapy. Eur J Pharmacol. (2025) 994:177376. doi: 10.1016/j.ejphar.2025.177376 39952582

[40] Aquino-Acevedo AN Knochenhauer H Castillo-Ocampo Y Ortiz-Leon M Rivera-Lopez YA Morales-Lopez C . Stress hormones are associated with inflammatory cytokines and attenuation of T-cell function in the ascites from patients with high grade serous ovarian cancer. Brain Behav Immun Health. (2022) 26:100558. doi: 10.1016/j.bbih.2022.100558 36439058 PMC9694096

[41] Abdel-Rasol MA El-Sayed WM . Nuclear receptors in metabolic, inflammatory, and oncologic diseases: mechanisms, therapeutic advances, and future directions. Eur J Med Res. (2025) 30:843. doi: 10.1186/s40001-025-03073-6 40926269 PMC12418679

[42] von Itzstein MS Gonugunta AS Wang Y Sheffield T Lu R Ali S . Divergent prognostic effects of pre-existing and treatment-emergent thyroid dysfunction in patients treated with immune checkpoint inhibitors. Cancer Immunol Immunother. (2022) 71:2169–81. doi: 10.1007/s00262-022-03151-2 35072744 PMC9308834

[43] Quan L Liu J Wang Y Yang F Yang Z Ju J . Exploring risk factors for endocrine-related immune-related adverse events: Insights from meta-analysis and Mendelian randomization. Hum Vaccin Immunother. (2024) 20:2410557. doi: 10.1080/21645515.2024.2410557 39377304 PMC11469449

[44] Kotwal A KottsChade L Ryder M . PD-L1 inhibitor-induced thyroiditis is associated with better overall survival in cancer patients. Thyroid. (2020) 30:177–84. doi: 10.1089/thy.2019.0250 31813343 PMC7047075

[45] Dong Y Li Y Peng X Fang W . Immune checkpoint inhibitors-induced thyroid dysfunction improves the prognosis of patients with lung cancer: a meta-analysis and systematic review. Front Endocrinol (Lausanne). (2025) 16:1743245. doi: 10.3389/fendo.2025.1743245 41641031 PMC12864112

[46] Basak EA van der Meer JWM Hurkmans DP Schreurs MWJ Oomen-de Hoop E van der Veldt AAM . Overt thyroid dysfunction and anti-thyroid antibodies predict response to anti-PD-1 immunotherapy in cancer patients. Thyroid. (2020) 30:966–73. doi: 10.1089/thy.2019.0726 32151195

[47] Okuji T Iwama S Kobayashi T Yasuda Y Ito M Yamagami A . Thyroid autoantibodies at baseline predict longer survival in non-small cell lung cancer patients treated with anti-programmed cell death-1 blockade: a prospective study. Nagoya J Med Sci. (2024) 86:452–63. doi: 10.18999/nagjms.86.3.452 PMC11439604 39355355

[48] De Pergola G Silvestris F . Obesity as a major risk factor for cancer. J Obes. (2013) 2013:291546. doi: 10.1155/2013/291546 24073332 PMC3773450

[49] Gubbels Bupp MR Jorgensen TN . Androgen-induced immunosuppression. Front Immunol. (2018) 9:794. doi: 10.3389/fimmu.2018.00794 29755457 PMC5932344

[50] Schwarzlmueller P Triebig A Assie G Jouinot A Theurich S Maier T . Steroid hormones as modulators of anti-tumoural immunity. Nat Rev Endocrinol. (2025) 21:331–43. doi: 10.1038/s41574-025-01102-2 40128599

[51] Smith LC Ramar M Riley GL 3rd Mathias CB Lee JY . Steroid hormone regulation of immunometabolism and inflammation. Front Immunol. (2025) 16:1654034. doi: 10.3389/fimmu.2025.1654034 41041336 PMC12483911

[52] Sabit H Adel A Abdelfattah MM Ramadan RM Nazih M Abdel-Ghany S . The role of tumor microenvironment and immune cell crosstalk in triple-negative breast cancer (TNBC): Emerging therapeutic opportunities. Cancer Lett. (2025) 628:217865. doi: 10.1016/j.canlet.2025.217865 40516902

[53] Zhang L Jiang T Yang Y Deng W Lu H Wang S . Postpartum hepatitis and host immunity in pregnant women with chronic HBV infection. Front Immunol. (2022) 13:1112234. doi: 10.3389/fimmu.2022.1112234 36685527 PMC9846060

[54] Festa A D'Agostino R Jr. Howard G Mykkanen L Tracy RP Haffner SM . Chronic subclinical inflammation as part of the insulin resistance syndrome: the Insulin Resistance Atherosclerosis Study (IRAS). Circulation. (2000) 102:42–7. doi: 10.1161/01.cir.102.1.42 10880413

[55] Lord GM Matarese G Howard JK Baker RJ Bloom SR Lechler RI . Leptin modulates the T-cell immune response and reverses starvation-induced immunosuppression. Nature. (1998) 394:897–901. doi: 10.1038/29795 9732873

[56] Wolf AM Wolf D Rumpold H Enrich B Tilg H . Adiponectin induces the anti-inflammatory cytokines IL-10 and IL-1RA in human leukocytes. Biochem Biophys Res Commun. (2004) 323:630–5. doi: 10.1016/j.bbrc.2004.08.145 15369797

[57] Trestini I Caldart A Dodi A Avancini A Tregnago D Sartori G . Body composition as a modulator of response to immunotherapy in lung cancer: time to deal with it. ESMO Open. (2021) 6:100095. doi: 10.1016/j.esmoop.2021.100095 33773420 PMC8027687

[58] McQuade JL Daniel CR Hess KR Mak C Wang DY Rai RR . Association of body-mass index and outcomes in patients with metastatic melanoma treated with targeted therapy, immunotherapy, or chemotherapy: a retrospective, multicohort analysis. Lancet Oncol. (2018) 19:310–22. doi: 10.1016/s1470-2045(18)30078-0 29449192 PMC5840029

[59] Kichenadasse G Miners JO Mangoni AA Rowland A Hopkins AM Sorich MJ . Association between body mass index and overall survival with immune checkpoint inhibitor therapy for advanced non-small cell lung cancer. JAMA Oncol. (2020) 6:512–8. doi: 10.1001/jamaoncol.2019.5241 31876896 PMC6990855

[60] Banack HR Stokes A . The 'obesity paradox' may not be a paradox at all. Int J Obes (Lond). (2017) 41:1162–3. doi: 10.1038/ijo.2017.99 28584251

[61] Rubino F Cummings DE Eckel RH Cohen RV Wilding JPH Brown WA . Definition and diagnostic criteria of clinical obesity. Lancet Diabetes Endocrinol. (2025) 13:221–62. doi: 10.1016/s2213-8587(24)00316-4 39824205 PMC11870235

[62] Santarpia L Marra M Montagnese C Alfonsi L Pasanisi F Contaldo F . Prognostic significance of bioelectrical impedance phase angle in advanced cancer: preliminary observations. Nutrition. (2009) 25:930–1. doi: 10.1016/j.nut.2009.01.015 19500944

[63] Neel DR McClave S Martindale R . Hypoalbuminaemia in the perioperative period: clinical significance and management options. Best Pract Res Clin Anaesthesiol. (2011) 25:395–400. doi: 10.1016/j.bpa.2011.07.006 21925404

[64] Quinlan GJ Martin GS Evans TW . Albumin: biochemical properties and therapeutic potential. Hepatology. (2005) 41:1211–9. doi: 10.1002/hep.20720 15915465

[65] Midik MM Gunenc D Acar PF Karaca BS . Prognostic value of blood-based inflammatory markers in cancer patients receiving immune checkpoint inhibitors. Cancers (Basel). (2024) 17:37. doi: 10.3390/cancers17010037 39796668 PMC11719015

[66] Zheng M . Serum albumin: a pharmacokinetic marker for optimizing treatment outcome of immune checkpoint blockade. J Immunother Cancer. (2022) 10:e005670. doi: 10.1136/jitc-2022-005670 36600664 PMC9772729

[67] Wang W Guo MN Li N Pang DQ Wu JH . Glutamine deprivation impairs function of infiltrating CD8(+) T cells in hepatocellular carcinoma by inducing mitochondrial damage and apoptosis. World J Gastrointest Oncol. (2022) 14:1124–40. doi: 10.4251/wjgo.v14.i6.1124 35949216 PMC9244988

[68] Zhang L Bonomi PD . Immune system disorder and cancer-associated cachexia. Cancers (Basel). (2024) 16:1709. doi: 10.3390/cancers16091709 38730660 PMC11083538

[69] Meng K Chen J Chen J Sun S Li H Zhou G . Risk factors for steroid-refractory in immune checkpoint inhibitor-induced colitis: a retrospective cohort study. Front Immunol. (2025) 16:1623150. doi: 10.3389/fimmu.2025.1623150 41126832 PMC12537882

[70] Proctor MJ Morrison DS Talwar D Balmer SM Fletcher CD O'Reilly DS . A comparison of inflammation-based prognostic scores in patients with cancer. A Glasgow Inflammation Outcome Study. Eur J Cancer. (2011) 47:2633–41. doi: 10.1016/j.ejca.2011.03.028 21724383

[71] Li J Yu L Hu X Huang T Chen M Zhang S . Usefulness of the preoperative Prognostic Immune and Nutritional Index as a prognostic predictor for patients with gastric cancer. Oncol Lett. (2025) 30:435. doi: 10.3892/ol.2025.15181 40688585 PMC12273764

[72] Khoo AL Chai LY Koenen HJ Sweep FC Joosten I Netea MG . Regulation of cytokine responses by seasonality of vitamin D status in healthy individuals. Clin Exp Immunol. (2011) 164:72–9. doi: 10.1111/j.1365-2249.2010.04315.x 21323660 PMC3074219

[73] Zhang Y Leung DY Richers BN Liu Y Remigio LK Riches DW . Vitamin D inhibits monocyte/macrophage proinflammatory cytokine production by targeting MAPK phosphatase-1. J Immunol. (2012) 188:2127–35. doi: 10.4049/jimmunol.1102412 22301548 PMC3368346

[74] BaSalamah MA Abdelghany AH El-Boshy M Ahmad J Idris S Refaat B . Vitamin D alleviates lead induced renal and testicular injuries by immunomodulatory and antioxidant mechanisms in rats. Sci Rep. (2018) 8:4853. doi: 10.1038/s41598-018-23258-w 29556070 PMC5859277

[75] Li P Li K Yuan W Xu Y Li P Wu R . 1alpha,25(OH)(2)D(3) ameliorates insulin resistance by alleviating gammadelta T cell inflammation via enhancing fructose-1,6-bisphosphatase 1 expression. Theranostics. (2023) 13:5290–304. doi: 10.7150/thno.84645 PMC10614678 37908738

[76] Fenercioglu AK . The anti-inflammatory roles of vitamin D for improving human health. Curr Issues Mol Biol. (2024) 46:13514–25. doi: 10.3390/cimb46120807 39727935 PMC11674702

[77] von Essen MR Kongsbak M Schjerling P Olgaard K Odum N Geisler C . Vitamin D controls T cell antigen receptor signaling and activation of human T cells. Nat Immunol. (2010) 11:344–9. doi: 10.1038/ni.1851 20208539

[78] Li P Zhu X Cao G Wu R Li K Yuan W . 1alpha,25(OH)(2)D(3) reverses exhaustion and enhances antitumor immunity of human cytotoxic T cells. J Immunother Cancer. (2022) 10:e003477. doi: 10.1136/jitc-2021-003477 35318258 PMC8943781

[79] Giampazolias E Pereira da Costa M Lam KC Lim KHJ Cardoso A Piot C . Vitamin D regulates microbiome-dependent cancer immunity. Science. (2024) 384:428–37. doi: 10.1126/science.adh7954 38662827 PMC7615937

[80] Galus Ł Michalak M Lorenz M Stoińska-Swiniarek R Tusień Małecka D Galus A . Vitamin D supplementation increases objective response rate and prolongs progression-free time in patients with advanced melanoma undergoing anti-PD-1 therapy. Cancer. (2023) 129:2047–55. doi: 10.1002/cncr.34718 37089083

[81] Braun N Baker N Peacker BL El Banna G Guggina L Reynolds KL . Vitamin D deficiency is associated with lower overall survival in patients receiving immune checkpoint inhibitors for cancer: A population-based cohort study using the TriNetX database. J Am Acad Dermatol. (2026) 94:663–6. doi: 10.1016/j.jaad.2025.10.037 41086959

[82] Reichrath J Biersack F Wagenpfeil S Schope J Pfohler C Saternus R . Low vitamin D status predicts poor clinical outcome in advanced melanoma treated with immune checkpoint or BRAF/MEK inhibitors: A prospective non-interventional side-by-side analysis. Front Oncol. (2022) 12:839816. doi: 10.3389/fonc.2022.839816 35669434 PMC9166268

[83] Kumar A Ye C Nkansah A Decoville T Fogo GM Sajjakulnukit P . Iron regulates the quiescence of naive CD4 T cells by controlling mitochondria and cellular metabolism. Proc Natl Acad Sci USA. (2024) 121:e2318420121. doi: 10.1073/pnas.2318420121 38621136 PMC11047099

[84] Deng J Liu N Zhao H Tian H Kikkawa A Tanaka K . Self-amplifying ROS-responsive trinity nanodelivery system for enhanced cancer therapy. Int J Pharm. (2025) 683:126091. doi: 10.1016/j.ijpharm.2025.126091 40840811

[85] Sacco A Battaglia AM Botta C Aversa I Mancuso S Costanzo F . Iron metabolism in the tumor microenvironment-implications for anti-cancer immune response. Cells. (2021) 10:303. doi: 10.3390/cells10020303 33540645 PMC7913036

[86] Lin Z Zou S Wen K . The crosstalk of CD8+ T cells and ferroptosis in cancer. Front Immunol. (2023) 14:1255443. doi: 10.3389/fimmu.2023.1255443 38288118 PMC10822999

[87] Wu Y Zhang K Jiang N Chen Z Sun X Zha H . Iron-fueled ferroptosis: a new axis for immunomodulation to overcome cancer drug resistance-from immune microenvironment crosstalk to therapeutic translation. Front Immunol. (2025) 16:1726210. doi: 10.3389/fimmu.2025.1726210 41607787 PMC12835310

[88] Wang W Green M Choi JE Gijon M Kennedy PD Johnson JK . CD8(+) T cells regulate tumour ferroptosis during cancer immunotherapy. Nature. (2019) 569:270–4. doi: 10.1038/s41586-019-1170-y 31043744 PMC6533917

[89] Kapralov AA Yang Q Dar HH Tyurina YY Anthonymuthu TS Kim R . Redox lipid reprogramming commands susceptibility of macrophages and microglia to ferroptotic death. Nat Chem Biol. (2020) 16:278–90. doi: 10.1038/s41589-019-0462-8 32080625 PMC7233108

[90] Xu C Sun S Johnson T Qi R Zhang S Zhang J . The glutathione peroxidase Gpx4 prevents lipid peroxidation and ferroptosis to sustain Treg cell activation and suppression of antitumor immunity. Cell Rep. (2021) 35:109235. doi: 10.1016/j.celrep.2021.109235 34133924

[91] Abdelhamed S Zhang X Zeng W Grogan B Schilperoort L James R . PF-08046032: A novel, investigational CD25-directed antibody-drug conjugate optimized for selective depletion of regulatory T cells in advanced Malignant tumors. Mol Cancer Ther. (2025) 24:963–75. doi: 10.1158/1535-7163.MCT-25-0101 40293942

[92] Li Y Cao Y Ma K Ma R Zhang M Guo Y . A triple-responsive polymeric prodrug nanoplatform with extracellular ROS consumption and intracellular H(2)O(2) self-generation for imaging-guided tumor chemo-ferroptosis-immunotherapy. Adv Healthc Mater. (2024) 13:e2303568. doi: 10.1002/adhm.202303568 38319010

[93] Tekeoglu I Sahin MZ Kamanli A Nas K . The influence of zinc levels on osteoarthritis: A comprehensive review. Nutr Res Rev. (2025) 38:282–93. doi: 10.1017/S0954422424000234 39311401

[94] Maywald M Wessels I Rink L . Zinc signals and immunity. Int J Mol Sci. (2017) 18:2222. doi: 10.3390/ijms18102222 29064429 PMC5666901

[95] Prasad AS . Clinical, immunological, anti-inflammatory and antioxidant roles of zinc. Exp Gerontol. (2008) 43:370–7. doi: 10.1016/j.exger.2007.10.013 18054190

[96] Beck FW Prasad AS Kaplan J Fitzgerald JT Brewer GJ . Changes in cytokine production and T cell subpopulations in experimentally induced zinc-deficient humans. Am J Physiol. (1997) 272:E1002–1007. doi: 10.1152/ajpendo.1997.272.6.e1002 9227444

[97] Maggini S Wintergerst ES Beveridge S Hornig DH . Selected vitamins and trace elements support immune function by strengthening epithelial barriers and cellular and humoral immune responses. Br J Nutr. (2007) 98:S29–35. doi: 10.1017/s0007114507832971 17922955

[98] Jarosz M Olbert M Wyszogrodzka G Mlyniec K Librowski T . Antioxidant and anti-inflammatory effects of zinc. Zinc-dependent NF-kappaB signaling. Inflammopharmacology. (2017) 25:11–24. doi: 10.1007/s10787-017-0309-4 28083748 PMC5306179

[99] Bao B Thakur A Li Y Ahmad A Azmi AS Banerjee S . The immunological contribution of NF-kappaB within the tumor microenvironment: a potential protective role of zinc as an anti-tumor agent. Biochim Biophys Acta. (2012) 1825:160–72. doi: 10.1016/j.bbcan.2011.11.002 22155217 PMC3811120

[100] Xu Y Yuan W Li K Li P . Nutritional intervention alleviates T cell exhaustion and empowers anti-tumor immunity. Front Immunol. (2025) 16:1689317. doi: 10.3389/fimmu.2025.1689317 41488640 PMC12757304

[101] Qiu Y Su Y Xie E Cheng H Du J Xu Y . Mannose metabolism reshapes T cell differentiation to enhance anti-tumor immunity. Cancer Cell. (2025) 43:103–21:e108. doi: 10.1016/j.ccell.2024.11.003 PMC11756673 39642888

[102] Cao T Zhang W Wang Q Wang C Ma W Zhang C . Cancer SLC6A6-mediated taurine uptake transactivates immune checkpoint genes and induces exhaustion in CD8(+) T cells. Cell. (2024) 187:2288–304:e2227. doi: 10.1016/j.cell.2024.03.011 38565142

[103] Shentu J Su X Yu Y Duan S . Unveiling the role of taurine and SLC6A6 in tumor immune evasion: Implications for gastric cancer therapy. Int J Biochem Cell Biol. (2024) 176:106661. doi: 10.1016/j.biocel.2024.106661 39270578

[104] Colucci M Zumerle S Bressan S Gianfanti F Troiani M Valdata A . Retinoic acid receptor activation reprograms senescence response and enhances anti-tumor activity of natural killer cells. Cancer Cell. (2024) 42:646–61:e649. doi: 10.1016/j.ccell.2024.02.004 PMC11003464 38428412

[105] Liu G Quan Q Pan L Duan H Zhang G Li K . Retinoic acid enhances gammadelta T cell cytotoxicity in nasopharyngeal carcinoma by reversing immune exhaustion. Cell Commun Signal. (2025) 23:156. doi: 10.1186/s12964-025-02161-8 40158172 PMC11955114

[106] Wiertsema SP van Bergenhenegouwen J Garssen J Knippels LMJ . The interplay between the gut microbiome and the immune system in the context of infectious diseases throughout life and the role of nutrition in optimizing treatment strategies. Nutrients. (2021) 13:886. doi: 10.3390/nu13030886 33803407 PMC8001875

[107] Xie Y Deng D Wang S Li Z Mo T Wang Y . Composite dietary fiber alleviates obesity-induced skeletal muscle atrophy by regulating gut microbiota-derived short-chain fatty acids in mice. NPJ Sci Food. (2026) 10:49. doi: 10.1038/s41538-025-00698-z 41513674 PMC12891578

[108] Yang W Yu T Huang X Bilotta AJ Xu L Lu Y . Intestinal microbiota-derived short-chain fatty acids regulation of immune cell IL-22 production and gut immunity. Nat Commun. (2020) 11:4457. doi: 10.1038/s41467-020-18262-6 32901017 PMC7478978

[109] Yan H Ajuwon KM . Butyrate modifies intestinal barrier function in IPEC-J2 cells through a selective upregulation of tight junction proteins and activation of the Akt signaling pathway. PloS One. (2017) 12:e0179586. doi: 10.1371/journal.pone.0179586 28654658 PMC5487041

[110] He Y Fu L Li Y Wang W Gong M Zhang J . Gut microbial metabolites facilitate anticancer therapy efficacy by modulating cytotoxic CD8(+) T cell immunity. Cell Metab. (2021) 33:988–1000:e1007. doi: 10.1016/j.cmet.2021.03.002 33761313

[111] Zhu X Li K Liu G Wu R Zhang Y Wang S . Microbial metabolite butyrate promotes anti-PD-1 antitumor efficacy by modulating T cell receptor signaling of cytotoxic CD8 T cell. Gut Microbes. (2023) 15:2249143. doi: 10.1080/19490976.2023.2249143 37635362 PMC10464552

[112] Dubin K Callahan MK Ren B Khanin R Viale A Ling L . Intestinal microbiome analyses identify melanoma patients at risk for checkpoint-blockade-induced colitis. Nat Commun. (2016) 7:10391. doi: 10.1038/ncomms10391 26837003 PMC4740747

[113] McCulloch JA Davar D Rodrigues RR Badger JH Fang JR Cole AM . Intestinal microbiota signatures of clinical response and immune-related adverse events in melanoma patients treated with anti-PD-1. Nat Med. (2022) 28:545–56. doi: 10.1038/s41591-022-01698-2 PMC10246505 35228752

[114] Gao YQ Tan YJ Fang JY . Roles of the gut microbiota in immune-related adverse events: mechanisms and therapeutic intervention. Nat Rev Clin Oncol. (2025) 22:499–516. doi: 10.1038/s41571-025-01026-w 40369317

[115] Kang X Lau HC Yu J . Modulating gut microbiome in cancer immunotherapy: Harnessing microbes to enhance treatment efficacy. Cell Rep Med. (2024) 5:101478. doi: 10.1016/j.xcrm.2024.101478 38631285 PMC11031381

[116] Davar D Dzutsev AK McCulloch JA Rodrigues RR Chauvin JG Morrison RM . Fecal microbiota transplant overcomes resistance to anti-PD-1 therapy in melanoma patients. Science. (2021) 371:595–602. doi: 10.1126/science.abf3363 33542131 PMC8097968

[117] Saghafi S Yaghoubi MA Safarpour H Raeisi H Mousavi Z . Protecting the endocrine axis in immuno-oncology: GLP-1 receptor agonists as host-directed modulators in colorectal cancer. BioMed Pharmacother. (2026) 194:118948. doi: 10.1016/j.biopha.2025.118948 41496347

[118] Michailidou D Khaki AR Morelli MP Diamantopoulos L Singh N Grivas P . Association of blood biomarkers and autoimmunity with immune related adverse events in patients with cancer treated with immune checkpoint inhibitors. Sci Rep. (2021) 11:9029. doi: 10.1038/s41598-021-88307-3 33907229 PMC8079370

[119] Sadashima E Hattori S Takahashi K . Meta-analysis of prognostic studies for a biomarker with a study-specific cutoff value. Res Synth Methods. (2016) 7:402–19. doi: 10.1002/jrsm.1201 26873370

[120] Schmidt M Loibl S . Chemotherapy in older patients with early breast cancer. Breast. (2024) 78:103821. doi: 10.1016/j.breast.2024.103821 39405593 PMC11752109

[121] You W Liu X Tang H Lu B Zhou Q Li Y . Vitamin D status is associated with immune checkpoint inhibitor efficacy and immune-related adverse event severity in lung cancer patients: A prospective cohort study. J Immunother. (2023) 46:236–43. doi: 10.1097/CJI.0000000000000469 37184520

[122] Caccialanza R Cereda E Agustoni F Klersy C Casirati A Montagna E . Multicentre, randomised, open-label, parallel-group, clinical phase II study to evaluate immunonutrition in improving efficacy of immunotherapy in patients with metastatic non-small cell lung cancer, undergoing systematic nutritional counseling. BMC Cancer. (2022) 22:1212. doi: 10.1186/s12885-022-10296-x 36434615 PMC9700895

[123] Yang J Wang C Xue J Ma X Hou Y Yuan H . Prognostic value of Prognostic Nutritional Index in cancer: an umbrella review of systematic review and meta-analysis. Front Nutr. (2026) 12:1706534. doi: 10.3389/fnut.2025.1706534 41648751 PMC12867775

[124] An S Kim S Eo W Lee S . The prognostic immune and nutritional index as a predictor of survival in resected non-small cell lung cancer. Med (Kaunas). (2025) 61:1763. doi: 10.3390/medicina61101763 PMC12565816 41155750

[125] Jing Y Ren M Li X Sun X Xiao Y Xue J . The effect of systemic immune-inflammatory index (SII) and prognostic nutritional index (PNI) in early gastric cancer. J Inflammation Res. (2024) 17:10273–87. doi: 10.2147/JIR.S499094 PMC11625636 39654858

[126] Cobbold SP . The mTOR pathway and integrating immune regulation. Immunology. (2013) 140:391–8. doi: 10.1111/imm.12162 PMC3839643 23952610

[127] Suzuki J Yamada T Inoue K Nabe S Kuwahara M Takemori N . The tumor suppressor menin prevents effector CD8 T-cell dysfunction by targeting mTORC1-dependent metabolic activation. Nat Commun. (2018) 9:3296. doi: 10.1038/s41467-018-05854-6 30120246 PMC6098065

[128] Sinclair LV Rolf J Emslie E Shi YB Taylor PM Cantrell DA . Control of amino-acid transport by antigen receptors coordinates the metabolic reprogramming essential for T cell differentiation. Nat Immunol. (2013) 14:500–8. doi: 10.1038/ni.2556 PMC3672957 23525088

[129] GLP-1s spur greater reduction in colorectal cancer risk than aspirin. Cancer Discov. (2026) 16:OF1. doi: 10.1158/2159-8290.CD-NW2026-0002

[130] Karakasis P Patoulias D Fragakis N Mantzoros CS . Effect of glucagon-like peptide-1 receptor agonists and co-agonists on body composition: Systematic review and network meta-analysis. Metabolism. (2025) 164:156113. doi: 10.1016/j.metabol.2024.156113 39719170

[131] Wilding JPH Batterham RL Calanna S Davies M Van Gaal LF Lingvay I . Once-weekly semaglutide in adults with overweight or obesity. N Engl J Med. (2021) 384:989–1002. doi: 10.1056/NEJMoa2032183 33567185

[132] Look M Dunn JP Kushner RF Cao D Harris C Gibble TH . Body composition changes during weight reduction with tirzepatide in the SURMOUNT-1 study of adults with obesity or overweight. Diabetes Obes Metab. (2025) 27:2720–9. doi: 10.1111/dom.16275 PMC11965027 39996356

[133] Jastreboff AM Aronne LJ Ahmad NN Wharton S Connery L Alves B . Tirzepatide once weekly for the treatment of obesity. N Engl J Med. (2022) 387:205–16. doi: 10.1056/NEJMoa2206038 35658024

[134] Memel Z Gold SL Pearlman M Muratore A Martindale R . Impact of GLP-1 receptor agonist therapy in patients high risk for sarcopenia. Curr Nutr Rep. (2025) 14:63. doi: 10.1007/s13668-025-00649-w 40289060

[135] Hoffmann C Weigert C . Skeletal muscle as an endocrine organ: the role of myokines in exercise adaptations. Cold Spring Harb Perspect Med. (2017) 7:a029793. doi: 10.1101/cshperspect.a029793 28389517 PMC5666622

[136] Kennedy MK Glaccum M Brown SN Butz EA Viney JL Embers M . Reversible defects in natural killer and memory CD8 T cell lineages in interleukin 15-deficient mice. J Exp Med. (2000) 191:771–80. doi: 10.1084/jem.191.5.771 PMC2195858 10704459

[137] Nelke C Dziewas R Minnerup J Meuth SG Ruck T . Skeletal muscle as potential central link between sarcopenia and immune senescence. EBioMedicine. (2019) 49:381–8. doi: 10.1016/j.ebiom.2019.10.034 PMC6945275 31662290

[138] Guzman-Prado Y Ben Shimol J Samson O . Sarcopenia and the risk of adverse events in patients treated with immune checkpoint inhibitors: a systematic review. Cancer Immunol Immunother. (2021) 70:2771–80. doi: 10.1007/s00262-021-02888-6 PMC10991997 33625531

[139] Zheng Y . Study design considerations for cancer biomarker discoveries. J Appl Lab Med. (2018) 3:282–9. doi: 10.1373/jalm.2017.025809 PMC6391721 30828695

[140] Sturgeon C . Standardization of tumor markers - priorities identified through external quality assessment. Scand J Clin Lab Invest Suppl. (2016) 245:S94–9. doi: 10.1080/00365513.2016.1210334 27542005

[141] Vermeersch P Frans G von Meyer A Costelloe S Lippi G Simundic AM . How to meet ISO15189:2012 pre-analytical requirements in clinical laboratories? A consensus document by the EFLM WG-PRE. Clin Chem Lab Med. (2021) 59:1047–61. doi: 10.1515/cclm-2020-1859 33554545

[142] Schuck RN Sekar V . Use of biomarkers in drug development for regulatory purposes. Clin Transl Sci. (2025) 18:e70377. doi: 10.1111/cts.70377 41035359 PMC12489174

[143] Chen G Lin L Mai Z Tang Y Zhang Q Chen G . Carrier-free photodynamic bioregulators inhibiting lactic acid efflux combined with immune checkpoint blockade for triple-negative breast cancer immunotherapy. ACS Nano. (2024) 18(30):19875–89. doi: 10.1021/acsnano.4c07213 39034461

